# Efficacy of physical activity in the adjunctive treatment of major depressive disorders: preliminary results

**DOI:** 10.1186/1745-0179-3-8

**Published:** 2007-07-09

**Authors:** Alessandra Pilu, Manlio Sorba, Maria Carolina Hardoy, Anna Laura Floris, Francesca Mannu, Maria Luisa Seruis, Claudio Velluti, Bernardo Carpiniello, Massimiliano Salvi, Mauro Giovanni Carta

**Affiliations:** 1 Department of Public Health, University of Cagliari, Via Porcell 4, 09100 Cagliari, Italy; 2 Orthopedic Clinic, University of Cagliari, Ospedale Marino, Cagliari, Italy; 3 Italian Society of Sport Psychiatry, Viale Merello 22, 09100, Cagliari, Italy

## Abstract

**Background:**

No controlled trials have evaluated the long term efficacy of exercise activity to improve the treatment of patients with Major Depressive Disorders. The aim of the present study was to confirm the efficacy of the adjunctive physical activity in the treatment of major depressive disorders, with a long term follow up (8 months).

**Methods:**

Trial with randomized naturalistic control. Patients selected from the clinical activity registries of the Psychiatric Unit of the University of Cagliari, Italy.

Inclusion criteria: female, between 40 and 60 years, diagnosis of Major Depressive Disorders (DSM-IV TR) resistant to the ongoing treatment.

Exclusion criteria: diagnosis of psychotic disorders; any contraindications to physical activity.

30 patients (71.4% of the eligible) participated to the study.

Cases: 10 randomized patients undergoing pharmacological treatment plus physical activity.

Controls: 20 patients undergoing only pharmacological therapy.

The following tools were collected from each patient by two different psychiatric physicians at baseline and 8 month after the beginning of exercise program: SCID-I, HAM-D, CGI (Clinical Global Impression), GAF.

**Results:**

The patients that made physical activity had their HAM-D, GAF and CGI score improved from T0 to T8, all differences were statistically significant. In the control group HAM-D, GAF and CGI scores do not show any statistically significant differences between T0 and T8.

**Limits:**

Small sample size limited to female in adult age; control group was not subject to any structured rehabilitation activity or placebo so it was impossible to evaluate if the improvement was due to a non specific therapeutic effect associated with taking part in a social activity.

**Conclusion:**

Physical activity seems a good adjunctive treatment in the long term management of patients with MDD. Randomized placebo controlled trials are needed to confirm the results.

## Background

Several reports indicate that physical activity can reduce the severity of symptoms in depressed patients and physical exercise is an established non-pharmacological form of treatment for depressive disorders [1].

Despite recent advancements in the pharmacological treatment of major depressive disorder (MDD), about half of patients who receive treatment with antidepressant medication do not achieve full remission of symptoms. There is evidence that exercise can reduce depressive symptomatology when used as a treatment for MDD [2]. Recent studies have emphasized the chronic nature of depressive disorder, and the need for endorsing the same treatment protocols used for other chronic disease, such as diabetes. But duration of treatment does not seem to affect long-term prognosis of patients with depression, once the drug is stopped. Despite treating depression effectively in the short-term, antidepressant drugs may worsen its course due to the increases in the risk of relapse/recurrence [3]. Treatment of depression by pharmacological means is likely to leave residual symptoms in most patients. Such symptoms produce impairment and are important risk factors for relapse [3]. Thus the research is still interested in complementary treatments that may improve residual symptoms of depression and in safe treatments available for long term therapy contrasting the risk of relapse/recurrence. For both kind of needs physical exercise may be a useful resource. However, no randomized controlled trials have evaluated the long term follow up of patients with MDD still being symptomatic after an adequate antidepressant therapy in the acute phase, undergoing exercise activity to improve the efficacy of treatment [2].

The aim of the present study was to confirm the efficacy of the adjunctive physical activity in the treatment of major depressive disorder of patients non responders or partially responders to ongoing pharmacological treatment, with a long term follow up (8 months).

## Methods

### Study design

Trial with randomized naturalistic controls.

### Study population

Patients were selected from the clinical activity registries of the Psychiatric Unit of the University of Cagliari, Italy.

The inclusion criteria were the following:

- Female gender

- Age between 40 and 60 years

- Diagnosis of Major Depressive Disorders (DSM-IV TR) [4].

-No responsiveness to at least 1 antidepressant at adequate doses (HAMD >13 after at least 2 months of pharmacological treatment)

Exclusion criteria were

- Diagnosis of psychotic disorders

-Comorbidity with psychiatric disorders other than Generalized Anxiety Disorder (GAD), Social Phobia (SP), Panic Disorder (PD) with or without Agoraphobia,

- Any contraindications to physical activity

- Diagnosis of neurological and orthopaedic disorders at the time of the study (e.g.: Multiple Sclerosis, Rheumatoid Arthritis, Amyotrophic Lateral Sclerosis, Stroke, radicular or medullary compression, joint fractures, joint surgery, acquired limitations of joint movement).

### Study protocol

Among a total of 42 eligible patients, 30 (71.4%) consented to participate to the study, and signed informed consent was obtained from everyone. Patients were randomized after stratification for comorbidity with anxiety disorders (N = 13) between 2 arms of treatment, in a 2:1 fashion.

• Group I (cases): 10 patients (4 with comorbidity with anxiety disorder, 1 with SP, 1 PD, 2 GAD) undergoing pharmacological treatment (6 SSRI, 3 SNRI, 1 NARI) plus physical activity.

• Group II (controls): 20 patients (9 with comorbidity with anxiety disorder, 2 with SP, 3 PD, 4 GAD) undergoing only pharmacological therapy (14 SSRI, 2 SNRI, 1 NARI, SSRI + Tricyclic antidepressant).

Patients were evaluated at baseline (time 0: before starting the physical activity program), and at 2, 4, 6 and 8 month after the beginning of exercise program (time 2, 4, 6 and 8). Diagnosis were carried out according to DSM-IV-TR [3] by means of SCID-I (Structured Clinical Interview for DSM-IV Axis I Disorders) [5] administered by trained psychiatrists. These preliminary results concern the comparison between time 0 and time 8 of the data collected by two different trained psychiatric physicians by the following tools: HAM-D (Hamilton Rating Scale for Depression) [6], CGI (Clinical Global Impression) [7] and GAF (Global Assessment of Functioning) [8].

### Study treatment

The physical activity program included two 60 minute lessons per week, held by skilled an instructor, with ISEF (Physical Education) diploma, Psychology degree and post-degree diploma in sport Psychopathology (MS).

Each session was set in three steps:

Step I: welcome and warming up (about 5 minutes)

Step II: physiological strengthening (about 50 minutes)

Step III: stretching, cooling down, goodbye (about 5 minutes).

Exercise was performed without machines during step I and III, and with machines during step II, standing in semicircle or in a circle including the instructor, in order to enhance social communication among the members of the working-group.

The second step (physiological strengthening) was made with cardio-fitness machines and it consisted of different phases: first of all every patient could choose a machine and during the exercise she could communicated with the others near or in front of her. After every exercise (extended about 4 minutes) they could change the machine and choose another one. The machines were in total 20 and they allowed different exercises for arms, legs, postural muscles strengthening. Every lesson was overseen by a physician and a psychologist.

## Results

Analysis of variance (ANOVA) was used to examine possible group differences from baseline and 8 months of physical treatment.

Table 1 presents the difference among HAM-D, GAF and CGI scores in the overall group from t0 and t8.

**Table 1:** Scores (mean and standard deviation for each scale)

	T0 cases (N = 10)	T8 cases (N = 10)	ANOVA 1 way T0 vs T8 cases DF 1, 18, 19	T0 controls (N = 20)	T8 controls (N = 20)	ANOVA 1 way T0 vs T8 controls DF 1, 38, 39
HAM-D	20.5 ± 7.1	T6 8.1 ± 5.2	F = 19.89 P < 0.0001	19.3 ± 5.7	16.7 ± 9.1	F = 0.28 P = 0.28
ANOVA 1 way cases vs controls DF 1, 28, 29				F = 0.25 P = 0.620	F = 7.60 P = 0.010	
GAF	60.0 ± 9.0	75.4 ± 11.1	F = 11.23 P < 0.004	63.6 +/- 8.4	63.0 ± 7.9	F = 0.05 P = 0.817
ANOVA 1 way cases vs controls DF 1, 28, 29				F = 1.26 P = 0.27	F = 12.51 P < 0.0001	
CGI	4.0 ± 1.3	1.9 ± 0.8	F = 18.93 P < 0.0001	3.5 ± 0.9	4 ± 1.2	F = 0.09 P = 0.76
ANOVA 1 way cases vs controls DF 1, 28, 29				F = 1.53 P = 0.227	F = 12.68 P < 0.0001	

The patients that made physical activity had their HAM-D, GAF and CGI score improved from T0 to T8, all differences were statistically significant. In the control group HAM-D score improved moderately but the difference between T0 and T8 do not reach the statistical significance, the GAF score remains the same that was at the starting point at the end of the trial, the CGI score moderately decreases without any statistical significant difference.

## Discussion

We found that patients with resistant depressive disorders to the ongoing treatment using physical activity as adjunctive treatment show a depressive (HAMD) and general psychopathological (CGI) improvement after a long term (8 months) physical activity. The global functionality as measured by the GAF also improved. Controls with only pharmacotherapy do not improve their HAMD, CGI, GAF scores during the 8 months of the trial. Thus physical activity seems a good adjunctive treatment in the long term management of patients with MDD.

Previous reports about the efficacy of physical activity in MDD patients [9-13] have not a long term follow up (more than 6 months) like our trial.

## Limits

Small sample size limited to female in adult age; control group was not subject to any structured rehabilitation activity or placebo so it was impossible to evaluate if the improvement was due to a non specific therapeutic effect associated with taking part in a social activity.

## Conclusion

Long term use of exercise provides additional health benefits for MDD patients. Randomized placebo controlled placebo trials with large sample size are needed to confirm the results.
